# Effectiveness, acceptability and potential harms of peer support for self-harm in non-clinical settings: systematic review

**DOI:** 10.1192/bjo.2021.1081

**Published:** 2022-01-17

**Authors:** Nada Abou Seif, Rayanne John-Baptiste Bastien, Belinda Wang, Jessica Davies, Mette Isaken, Ellie Ball, Alexandra Pitman, Sarah Rowe

**Affiliations:** Division of Psychiatry, University College London, UK; Division of Psychiatry, University College London, UK; Division of Psychiatry, University College London, UK; St Andrews, Birmingham, UK; Samaritans, Surrey, UK; Samaritans, Surrey, UK; Division of Psychiatry, University College London, and Camden and Islington NHS Foundation Trust, UK; Division of Psychiatry, University College London, UK

**Keywords:** self-harm, self-injury, peer support, online forums, literature review

## Abstract

**Background:**

Many people who have self-harmed prefer informal sources of support or support from those with lived experience. However, little is known about whether peer support improves outcomes for people who have self-harmed or about the risks of peer support interventions in non-clinical settings.

**Aims:**

The aims of this review were to examine the effectiveness, acceptability and potential risks of peer support for self-harm, and how these risks might be mitigated.

**Method:**

We searched bibliographic databases and grey literature for papers published since 2000. We included peer support for self-harm that occurred in voluntary-sector organisations providing one-to-one or group support, or via moderated online peer support forums.

**Results:**

Eight of the ten papers included focused on peer support that was delivered through online media. No study compared peer support with other treatments or a control group, so limited conclusions could be made about its effectiveness. Peer support for self-harm was found to be acceptable and was viewed as having a range of benefits including a sense of community, empowerment, and access to information and support. The most commonly perceived risk associated with peer support was the potential for triggering self-harm.

**Conclusions:**

Our findings highlighted a range of benefits of being part of a group with very specific shared experiences. Mitigations for potential risks include organisations using professional facilitators for groups, trigger warnings for online forums, and providing regular supervision and training so that peers are prepared and feel confident to support vulnerable people while maintaining their own emotional health.

Self-harm is an act in which an individual initiates behaviour (such as self-cutting or ingesting a toxic substance or object) with the intention of causing harm to themselves with a non-fatal outcome.^1^ Definitions of self-harm vary according to the degree of suicidal intent, and it is important to note that not all people who practise self-harm feel suicidal when they self-harm.^2^ Self-harm is common, particularly in young people, and its prevalence is increasing in many countries around the world.^3–5^ Among British 17-year-olds, an estimated 20% of males and 28% of females report self-harm, with White and sexual minority adolescents identified as at particular risk.^6^ Self-harm is associated with distress and is the strongest risk factor for suicide.^7^ Many young people who self-harm prefer informal sources of support,^8^ or support from those with lived experience available through voluntary-sector organisations^9^ and online forums.^10^

Peer support interventions are increasingly adopted worldwide within mental health services and third-sector organisations (voluntary organisations, community organisations and charities).^11^ Although studies of peer support for mental health problems suggest that it is associated with positive effects on hope, recovery and quality of life, the effects on other outcomes such as symptoms, hospital admission and satisfaction are inconclusive.^12^ Peer support may have different benefits and harms depending on the population and setting in which it is used. Peer-reviewed research on peer support for people that self-harm is lacking, and little is known about the provision, quality, effectiveness and acceptability of these resources for young people or adults, or about the potential harms. The focus on peer support for self-harm, rather than wider mental distress, is important given the concerns about its appropriateness, particularly in the context of evidence describing imitative effect, and our clinical awareness of its relatively common usage. This review is therefore a unique contribution to the literature and will be clinically useful for those who care for people who self-harm.

In this systematic review of the quantitative and qualitative literature, we aimed to address the following research questions.

- What evidence exists for the availability and effectiveness of peer support for self-harm?
- What evidence exists on the risks of peer support for self-harm?
- What evidence exists on how the risks identified through the review could be mitigated?

## Method

Our systematic review followed PRISMA guidelines and was registered on the PROSPERO international prospective register of systematic reviews (CRD42021235441).

For the purpose of this review and in consultation with Samaritans, we used the term ‘self-harm’ to describe self-harm behaviour where there was no suicidal intent, ‘suicide attempts’ where there was suicidal intent, and ‘self-harm/suicide attempts’ where both applied or where this was unspecified or unclear. This was because we wanted to define the client group of interest very tightly, recognising that the provision, effectiveness, acceptability and potential harms of peer support for suicidal self-harm are likely to differ from those of support provided for non-suicidal self-harm. We also defined ‘peer support’ for self-harm as any support provided in non-clinical settings by individuals with lived experience of self-harm. We excluded peer support within clinical settings, as a recent parliamentary inquiry recommended investment in community-based preventive services, including low-level preventive support based on peer support models.^13^ We also excluded peer support provided by relatives or friends who self-harm, or peer support from people posting content about self-harm on the internet solely in a personal capacity, as these models would not benefit from funding.

Our definition therefore included: peer support provided by individuals working for voluntary-sector organisations (but not formal healthcare services) providing one-to-one or group support, or via moderated online peer support forums.

### Searches

Searches were conducted on MEDLINE (Ovid) <1946 to 15 February 2021> and PsycINFO (Ovid) <1806 to February week 2 2021>. Search terms were developed with the input of a lived experience researcher (J.D.) and in collaboration with the Samaritans team (E.B. and M.I.). Search terms covered keywords relevant to self-harm and both online and face-to-face peer support. These were combined into a single search string using the appropriate Boolean operators (see Supplementary material 1 available at https://doi.org/10.1192/bjo.2021.1081).

We also conducted searches for grey literature on both Open Grey and Google using the approach suggested in recent scoping reviews.^14^ For the Google searches, we made an *a priori* decision to screen only the first 100 results to reflect a balance of the relevance and time taken to screen each hit.^15^

We searched websites for UK charity organisations including Mind and Harmless and contacted each organisation to request any relevant publications on peer support. The list of organisations was composed based on suggestions by Samaritans and our team's lived experience researcher (Supplementary material 2).

Studies were included if they:

- described the provision, quality, effectiveness and acceptability of peer support for self-harm;
- related specifically to self-harm, regardless of suicidal intent;
- were published in English;
- were published from the year 2000 onwards;
- used quantitative, qualitative or mixed methods.

We did not set any restrictions on age group, population, study design or whether publications were peer reviewed. We also included studies with or without a comparator group, as we felt this was particularly important for capturing the acceptability of the peer support intervention. Social media sites that are moderated (for example, Reddit, Instagram and Twitter) were included in the review. Although their moderation may be considered more informal than other moderated forums, such as members-only forums, moderation still does take place. In addition to algorithms to identify harmful images or hashtags,^16^ social media platforms including Instagram and Twitter have tightened their policies regarding self-harm content. Graphic images related to self-harm are no longer allowed, and self-harm content is scrutinised by content moderators before being displayed or removed, thus making it closer to the formal moderation we would see in other online forums.^17^ Moderation on Reddit is primarily done by volunteers of individual subreddits known as “mods”, which is similar to member-only forums.^18^ We thought it would be useful to include both types of moderation, given the limited research on the subject and the high level of engagement with social media sites.

We excluded studies that:

- focused on suicide prevention without investigating self-harm specifically;
- described peer support in clinical settings;
- described peer support provided by relatives or friends;
- described peer support from people posting content on the internet solely in a personal capacity;
- peer support taking place on unmoderated online media (e.g. unmoderated forums).

### Data extraction

#### Screening and selection of studies

We used the Covidence systematic review online software (www.covidence.org) to import references from our search engines for screening titles and abstracts, and to deduplicate. Two reviewers screened all titles and abstracts independently. Four reviewers screened the full-text articles independently to determine their suitability for inclusion, and a randomly selected 10% of these underwent a second screening by an independent reviewer. Reference lists of included papers and relevant systematic reviews were checked for relevant papers. Any disagreement between the reviewers over the eligibility of studies was reviewed by third and fourth reviewers (S.R. and A.P.) and resolved through discussion. We calculated interrater agreement at each stage of the review screening process to assess the consistency of raters’ decisions. We used the accepted value of 0.8 as the threshold for good interrater agreement,^19^ resolving screening disagreements where values fell below 0.8 through discussions with third and fourth reviewers.

#### Data extraction

Information on the following variables were extracted from all of the papers: study titles, authors, study type, country of origin, year of publication, population, demographics (including age, sex and ethnicity), type of self-harm (suicidal, non-suicidal, both or not specified) details of the peer support intervention (nature, description, duration source of provision), outcome measures, change scores or themes relevant to the peer support intervention, risks or harmful effects, and mitigation of risks or harmful effects. A second reviewer independently checked the data extraction.

The outcome measures in relation to each of our review questions were as follows.

- Mean reduction in self-harm behaviours post peer support intervention.
- Changes in mean questionnaire scores, or themes relevant to risks or harmful effects of self-harm peer support interventions as derived from qualitative research.
- Ways in which the risks identified in research question (b) might be mitigated, as established using the results and discussion sections of all included papers.

#### Quality appraisal

One reviewer independently assessed the quality of each included paper, and a randomly selected subsample of 10% of included papers was independently assessed for study quality by a second researcher.

For quantitative peer-reviewed published papers, we used the Grading of Recommendations, Assessment, Development, and Evaluation (GRADE), which rates papers according to five domains: risk of bias, imprecision, inconsistency, indirectness and publication bias. A certainty/quality rating is assigned to the evidence, ranging from very low (the true effect is likely to be substantially different from the estimated effect) to high (we are confident that the effect of the study reflects the actual effect).^20^

For qualitative studies we used the Critical Appraisals Skill Programme (CASP) Qualitative Checklist, which examines whether there is sufficient description and justification of the chosen methods of data collection, sampling and analytical approach, as well as whether sufficient attention was given to ethics and the roles of the researchers involved.^21^

For studies using a mixed method design, we used the Mixed Methods Appraisal Tool (MMAT) version 2018,^22^ which includes a checklist to appraise the methodological quality for qualitative, quantitative and specifically mixed methods studies. MMAT examines whether the rationale of using a mixed methods design is appropriate and whether the different components of the study are incorporated constructively to answer the research question.

For non-academic papers (including grey literature), we used Nesta's Standard of Evidence model to assess the quality of each source of information.^23^ This considers criteria such as ‘Have others proved the same?’ and ‘Can this be replicated elsewhere?’ to judge whether the innovation described has evidence of benefits or harms.

#### Data synthesis

Anticipating a heterogeneous range of papers, we used a narrative synthesis to summarise themes relevant to our review questions. In team discussions, which included Samaritans team members and our lived experience researcher, we explored reflexivity in our interpretation of the findings to ensure that our inferences regarding recommendations for practice were appropriate, acceptable and relevant.

## Results

**Fig. 1:**
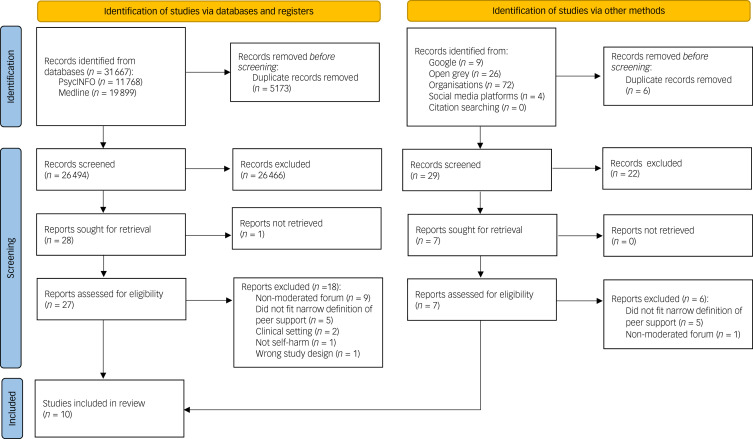
PRISMA flow diagram.

Our MEDLINE and PsycINFO searches identified 31 667 records, with an additional 35 records identified through OpenGrey and Google searches (Fig. 1). After the removal of duplicates, a total of 26 523 titles and abstracts were screened. Of those, 28 full-text articles were assessed for eligibility, of which a total of nine studies were identified as eligible for inclusion in our final synthesis. A further 35 records were identified from non-profit organisations, which we reduced to seven following deduplication and screening of titles and abstracts. After full-text screening, one of these records was judged to meet eligibility criteria for inclusion in our final analysis based on its specific focus on online peer support for young people self-harming.^24^ Other records from non-profit organisations were excluded owing to peer support being offered for difficulties not limited to self-harm or for not fitting our description of a peer support intervention. Interrater agreement was high for screening titles and abstracts (99%) but decreased to 75% for the full-text screening. One paper required discussion with a third reviewer before a consensus could be reached on its inclusion in the review and an interrater agreement greater than 0.8 could be achieved.

### Description of studies

Of the ten studies included in our narrative synthesis, eight were conducted in the UK and one in the USA, and one paper combined data from a range of mental health organisations in the UK, Italy, Slovenia and Denmark.^24^ Of the included studies, eight were of qualitative design and two used mixed methods (Tables 1 and 2). Notably, no randomised controlled trial or other trial investigating effectiveness met our inclusion criteria. In eight of the studies, peer support was delivered through online media, such as self-harm forums or message boards (*n* = 5), online recovery groups (*n* = 1), social media (*n* = 1), or a variety of platforms, including group chats, online group discussions and Facebook groups (*n* = 1). The remaining two studies examined face-to-face self-harm recovery/support groups.

**Table 1:** Qualitative studies and reports included in main results

Qualitative studies							
Study	Country	Population	Sample size and characteristics	Type of self-harm	Type of peer support	Key findings/themes	Perceived risks and mitigation
Boyce et al (2018)^32^	UK	Members of self-harm self-help groups; in-depth semi-structured interviews	*N* = 8 Mean age: 46 years Gender: seven female, one male Majority started self-harming as teenagers, with two members starting in late 20 s/early 30s Four members were previously in-patients (two ‘heard voices’, two treated for alcohol and drug dependence) One member said all members of the first group suffered from depression, but the other members did not explicitly state this	Not specified	Type: face to face, group Description: Two self-harm self-help groups. One of the groups also had a 24 h crisis support mobile telephone line and held a number of social activities. Sources and recipients of peer support: other members	Five themes: (a) Members’ experiences prior to joining the groups (negative experiences of isolation, stigma and shame) (b) A safe space (viewed the group as a safe space, shared experiential understanding, peer support felt more heartfelt and genuine) (c) A different approach (group A rejected stigmatising labels, group B resisted completing monitoring outcomes, neither group was focused on the cessation of self-harm but more on ‘managing a bit better’) (d) Alleviation of isolation (e) Learning from others	None identified
Brow (2015) ^25^	USA	Young adult users of self-injury message boards, analysis of text from online injury message boards	*N* = 10 Age: 18–24 years Gender: eight female, one male, one genderqueer Ethnicity: seven White, two Latina, one African American Participants began self-harming between the ages of 10 and 20 Nine (90%) participants had seen a counsellor or therapist Five (50%) had seen a therapist Four (40%) were currently seeing a therapist	Non-suicidal self-injury	Type: online Description: self-injury message board (11 of the 13 were moderated) Sources and recipients of peer support: users of the message board	Five themes identified: (a) Feeling less alone (b) Support from people who understand self-injury (c) Access to a community (d) Boards prompted seeking in-person support (e) Increased motivation to recover Perceived effect on self-injury No participant felt their self-injury was made worse (more frequent or severe) One (10%) participant reported being triggered to self-harm once (on an unmoderated board) Nine participants (90%) noted positive effects on their level of self-injury, relating to feeling increased support from board members Five participants (50%) recalled specific instances when their board use prevented self-harm	Risk: Risk of triggering board members Mitigation: Clinicians should check with their clients whether they use these online boards and, if so, whether they have ever been triggered
Corcoran et al (2007)^29^	UK	Women in self-injury support groups; semi-structured interviews	*N* = 7 Age: 21–44 years (mean = 36 years) Gender: 100% female Ethnicity: 100% White ‘All had current contact with professional services regarding self-injury and/or associated difficulties’	Not specified	Type: face to face, group Description: three city-based support groups Sources and recipients of peer support: group members	Four themes: (a) Belonging (i) Acceptance (ii) Safety (b) Sharing (sharing experiences) (i) Perspective (ii) Giving/receiving support (c) Autonomy (a sense of autonomy from leading and running the group themselves) (i) Direction (ii) Control (d) Positive feeling (‘anticipation about the group, improved mood and light-heartedness, particularly in relation to their self-injury’) (i) Friendship (ii) Inspiration (empowerment and self-worth through witnessing and supporting others’ struggles and successes) (e) Change (participants associated group membership with change/development) (i) Individual change (ii) Interpersonal change	Risks: Members being re-traumatised by others’ past experiences or encouraged to self-injure following exposure to alternative methods of self-injury Mitigations: (a) Having a facilitator for the group, particularly a professional ‘in the background’ to help mediate difficult situations (e.g. leader burnout, critical/dominant members) (b) Establishment of clear and consistent boundaries (c) Supplementing with additional individual support
Haberstroh & Moyer (2012)^31^	USA	Members of an online self-injury recovery group; analysis of free text responses to an online survey	N = 17 Mean age: 36 years Gender: 17 female, 3 male Ethnicity: 100% White Average duration of self-injuring: 20 years (s.d. = 8)	Not specified	Type: online Description: moderated online self-harm recovery group Sources & recipients of peer support: members of the group	Themes: (a) Self-injury as a relationship (self-injury viewed as a friend and a constant companion) (b) Self-injury as emotional expression and comfort (self-injury provided an outlet to express painful feelings) (c) The online group supplemented counselling (participants viewed it as an additional source of support, and referred to poor treatment and stigma from traditional providers) (d) Online group support, connection, and feedback (therapeutic aspects of the group included feeling understood, giving and receiving feedback, and feeling less alone and isolated) Subthemes: Supportive understanding, relational connections, supportive feedback (e) Safety and frustration with the no-triggering norm (members were asked to refrain from sharing triggering material, which increased safety in the group, but the limitation on communication also caused some members frustration) (f) Asynchronous group limitations (delays in communication due to the use of moderated email communication – members felt chat rooms were and could be helpful)	Risks: (a) (i) The content of the group discussions may be triggering for members (ii) Unmoderated groups may be triggering (b) Potentially triggering for moderators who would have to implement the ‘no triggering norm’. Mitigation: (a) (i) Establishing overt ‘no triggering norms’ (members aren't allowed to share explicitly triggering content such as pictures) to provide structure and safety to the group to avoid triggering members. (ii) Moderating groups (b) Moderators should be people with established recovery and social support
Lavis & Winter (2020)^26^	UK	Young people, who use or have used social media (Twitter, Reddit, Instagram) to engage with self-harm content; analysis of online content	Ethnography: *N* = N/A Age: estimated to be 10-24 years Gender: not stated Semi-structured interviews: *N* = 10 Age: 18 + years Gender: 100% female	Not specified	Type: Online Description: engagement with self-harm content on social media (Instagram, Twitter and Reddit all moderated in different ways) Sources and recipients of peer support: users of the social media platforms	(a) From offline to online: motivations for seeking self-harm content on social media Included content relating to parental and professional support being seen as ‘threats’, the desire for professional support but lack of accessibility, support from other users on how to get help. A clear motivation was gaining support of other young people with shared experiences (b) Online interactions: giving and receiving peer support (c) From online to offline: the value, impact and ambivalence of peer support (d) Very valuable to young people, can potentially save a young person's life during crises. (e) Can feel like you need to keep showing that you need help (f) Content on ‘how to self-harm safely’ (g) Congratulations when someone resists self-harm	Risks: (a) Can disincentivise offline help-seeking (b) Users may feel the need to post ‘increasingly graphic textual or visual content’ to sustain peer support (competitive element, but out of a ‘desperate dynamic of need’) (c) Can be burdensome for those listening to others’ distress and potentially triggering (d) Can be potentially triggering to those listening to another's distress, negatively affecting their mental health (e) Can isolate people and make them think they're the only people who can understand (barrier to seeking offline support) Mitigation: Online peer support should include signposting to offline resources.
Murray & Fox (2006)^30^	UK	Members of an online self-harm discussion group; analysis of free text responses to an online survey	*N* = 102 Age: 12–47 years (mean = 21.4 years) Gender: 95 female, six male, one declined to mention Mean age at which they began self-harm = 13.6 years	Not specified	Type: online, self-harm discussion group Description: moderated self-harm discussion group Sources and recipients of peer support: group members	Responses to closed questions: 42% reported feeling less alone 33% reported feeling understood 31% could not imagine a time when they would not need to use the discussion 41% reported a decrease in self-harm 46% reported no change in self-harm 10% reported an increase in self-harm 48% reported being triggered to self-harm by content of posts 47% reported never being triggered to self-harm by content of posts Open ended questions: 37% chose to talk online about self-harm to receive support from like-minded people (*n* = 99) 44% believed a decline in self-harm would result in a decrease in discussion group use (*n* = 79) 20% would want to continue membership in order to help others (*n* = 79) 48% believed being a member has reduced their self-harm (*n* = 79) 10% believed being a member has had other positive effects (e.g. more self-understanding) (*n* = 79) 32% felt there were no posts that triggered their self-harm (*n* = 74) 11% reported wanting to be triggered (*n* = 74) 45% reported ‘friendship’ as the reason for using private e-mail rather than the discussion group.	Risks: The impact on self-harm may be dependent on the ‘culture’ of the group/forum Mitigation: none identified
Sharkey et al (2012)^28^	UK	Members of an online self-harm support forum; analysis of online content	*N* = 77 young people who self-harm; 18 National Health Service professionals and healthcare students Age: 16–25 years 61% (*n* = 47) aged <20 years Gender: not stated	Not specified	Type: online Description: moderated self-harm support forum (SharpTalk) developed with input from young people who have self-harmed Sources of peer support: other members of SharpTalk (including young people who have self-harmed and professionals) Recipients of peer support: members of SharpTalk	Use of mitigation devices/strategies when giving advice online: Disclaimers/statement of incompetence (e.g. ‘if I'm not wrong’; ‘unless I'm mistaken’) –Hedges (maybe, sort of, somewhat, etc.) -Tag questions as indirect advice-giving (e.g. ‘do you think…?’) -Protective line (i.e. being polite to avoid conflict)	Risks: none identified Mitigation: Use of mitigation devices as face-work may help young people ‘do relationships’ within the context of the online support group to help to build rapport, minimise imposition and stay online
Smithson et al (2011)^27^	UK	Members of an online self-harm support forum; analysis of online forum content	*N* = 77 young people who have self-harmed; Six moderators Age and gender: not stated	Not specified	Type: online Description: moderated self-harm support forum (SharpTalk) developed with input from young people who have self-harmed Sources of peer support: other members of SharpTalk (including young people who have self-harmed and professionals) Recipients of peer support: members of SharpTalk	Themes: (a) Becoming members by displaying expectations about ways of discussing self-harm, and about responses and advice (b) Sustaining membership and setting boundaries of membership by shaping site in line with their expectation of how it should operate (c) Boundaries of membership: perceived deviance in posting behaviour and giving of healthcare advice were most commonly addressed by other users rather moderators (d) Feeling accepted and ‘belonging’ on the forum compared with bigger sites	Risks: Some posters struggled to maintain appropriate boundaries and avoid triggering or responding to triggers from other young people who have self-harmed Mitigation: Understanding the ways of posting and being accepted on a site, how young people achieve membership of an online support forum and feel ‘belonging’ may enable moderators to identify and support vulnerable participants who struggle to display appropriate member behaviour

**Table 2:** Mixed methods studies and reports included in main results

Mixed methods studies								
Study	Country	Population	Sample size and characteristics	Type of self-harm	Types of peer support	Outcome measures	Key findings	Risks and mitigation
Institute for Research and Development (UTRIP) (2012)^24^	UK, Italy, Slovenia, Denmark	Young people who have self-harmed; online surveys of users of peer support and professional s involved in self-harm support; analysis of online content; face-to-face interviews, phone interviews, interviews by email and by online chats; analysis of search terms used for self-harm support	YouthNet, UK: *N* = 429 Age: 16–25 years Gender: 299 females, 126 males Photofficine, Italy: Content feedback survey: *N* = 43 Pre-counselling survey: *N* = 94 Post-counselling survey: *N* = 70 Ongoing group support survey: *N* = 22 Post group support survey: *N* = 12 Age:12–25 years Gender: Content feedback survey: 38 females, 5 males Pre-counselling survey: 88 females, 12 males Post-counselling survey: 67 females, 3 males Ongoing group support survey: 21 females, 11 males Post group support survey: 11 females, 1 male Cyberhus, Denmark: Content feedback survey: *N* = 40 Pre group chat survey: *N* = 24 Post group chat survey: *N* = 24 Age: 12–21 years UTRIP, Slovenia: Survey for doctors in primary care for children and young people (CYP): *N* = 129 Survey for school counsellors, P.E. teachers, social workers, doctors and nurses: *N* = 294 Survey for young people who have self-harmed and their peers: *N* = 92 Age: 16–25 years Gender: UTRIP, Slovenia: Survey for doctors in primary care for CYP: N/A Survey for school counsellors, P.E. teachers, social workers, doctors and nurses: 256 females, 38 males Survey for young people who have self-harmed and their peers: 76 females, 16 males	Not specified	Type: online group chats, online group discussions, Facebook groups Sources and recipients of peer support: other users	N/A	(a) Easy and quick access to information UK: 53% of young people recognised the ease and speed of finding information as a benefit of online services, and 49% recognised the opportunity to access info whenever needed Italy: 66% of surveyed users recognised that when they had issues they needed help with, they received timely assistance, advice and information online (b) Connecting with others Percentage of young people feeling connected to others: YouthNet (UK): 88% Photofficine (Italy): 73% Cyberhus (Denmark): 67% Percentage of young people feeling supported by services offered: (i) People behind the service listened, took me seriously and cared about my problem: YouthNet (UK): 75% Photofficine (Italy): 60% Cyberhus (Denmark): 60% (ii) People using the service listened, took me seriously and cared about my problem: YouthNet (UK): 72% Photofficine (Italy): N/A Cyberhus (Denmark): 65% (iii) Safe anonymous space Percentage of young people feeling the organisation is a place to share their experiences: YouthNet (UK): 89% Photofficine (Italy): 71% Cyberhus (Denmark): 66%	Risks: (a) Triggering arguments (b) Being asked to help could be overwhelming (c) Young people don't always know how to help Mitigation: (a) Be aware of the potential of triggering, and moderate appropriately (b) Important that the young person offering help/being asked to help is in a safe and emotionally strong space themselves (c) In some cases, there is a need for professional input
Whitlock et al (2006)^33^	USA	Users of an online self-harm message board; analysis of online content on message boards	*N* = not stated Age: Site A: 18.7 (12–44) years; Site B: 18.3 (13–54) years, Site C: 19.4 (14–47) years; Site D: 17.6 (12–37) years; Site E: 17.5 (14–22) years; Site F: 19.6 (14–36) years; Site G: 20.5 (16–47) years; Site H: 18.1 (14–28) years; Site I: 23.9 (15–46) years; Site J: 16.4 (13–26) years	Not specified	Type: online Description: moderated message boards and individual posts. Message boards varied in moderation level from high (content blocked if triggering or graphic) to medium (content labelled as triggering). Focused on low to medium moderation Sources and recipients of peer support: other users	Message board analysis: date of establishment, number of active and inactive members, percentage of posters with blogs, co-listing with message boards for other behaviours	Quantitative Discouraging disclosure correlated with sharing techniques and seeking advice on stopping Seeking advice on stopping correlated with offers of informal support Suggesting formal treatment correlated with offering informal support and encouraging disclosure Qualitative: 11 themes identified: informal support and exchange, motivation for self-injury, concealment of self-injury behaviour, e.g. anxiety about exposure, methods for concealment of cuts and scars, addiction language (e.g. days of being self-injury free, difficulty stopping), formal help-seeking and treatment, sharing techniques, links to other mental health or behavioural conditions known to be associated with self-injurious behaviour, references to popular culture, perceptions of non-self-injurers reactions to self-injurious behaviour, perception of self and behaviour (e.g. self-worth, lovability, dissociation) and venting or apologising	Risks: There was a positive correlation between discussion of technique sharing with negative attitudes towards disclosure. Negative attitudes towards formal and informal help-seeking may reduce the use of alternative ways of coping Participation in self-injury message boards may expose adolescents to a culture where self-injury is normalised and encouraged, such as concealing self-injurious behaviours and sharing of techniques Adolescents’ drive to belong a group may lead to self-injurious behaviours

As per our inclusion criteria and research focus, all studies focused on individuals who self-harm, with five studies focusing specifically on young people and/or young adults who self-harm.^24–28^ Studies included a wide range of sample sizes, ranging from *n* = 7^29^ to *n* = 102^30^ participants. However, the report by the Institute for Research and Development (2012)^24^ was an outlier, as they included a range of samples sizes, and it was not clear whether they used the same participants in different stages of their surveys (Table 2). Although most studies did not report ethnicity, where ethnicity was reported (*n* = 3), individuals self-defining as White constituted 100% of the sample in two studies^29,31^ and 70% in one other.^25^ Females made up at least 80% of the samples across all studies. The majority of studies investigating online peer support sampled individuals within a young age range (16 to 25 years), apart from Haberstroh & Moyer (2012),^31^ in which the sample had a mean age of 36 years. The two studies investigating face-to-face peer support had samples with mean ages of 36 and 46 years, respectively.^29,32^

### Quality of included studies

All eight qualitative studies included were judged to be of high quality, scoring 8 or above out of 10 on the CASP checklist (Supplementary material 3A). However, studies tended to be unclear on how they addressed the relationship between the researchers and participants. Nevertheless, the other CASP domains were judged to be addressed adequately in most studies. Of the two mixed methods studies, one^33^ was scored as 11 out of 15 on the MMAT (as used for mixed methods research), with the domains for qualitative design judged as well addressed but those for quantitative design less so (Supplementary material 3B). The other mixed methods study^24^ was appraised using Nesta (as used for grey literature) and only rated as level 2, as the lack of comparator groups meant that the effects of the intervention could not be separated from other influences (Supplementary material 3C).

## Findings

The ten included studies identified a range of views on the acceptability and perceived value of peer support for self-harm, from both the perspective of those using the service and that of those providing it, along with descriptions of the mode of provision (Tables 1 and 2). We did not identify any study reporting the effectiveness of peer support for self-harm, nor any study presenting an overview of the provision of peer support for self-harm nationally or internationally.

### Q1. Mode of peer support, acceptability and effectiveness

#### Face-to-face peer support

Both the studies evaluating face-to-face peer support focused on self-harm support groups, and both reported on the experiences of members of more than one support group. Participants in the Boyce et al (2018) study discussed how members’ experiences prior to joining the group had been primarily negative and characterised by isolation, stigma and shame.^32^ Conversely, participants in both studies viewed their self-harm support groups as safe spaces, where they felt accepted and understood (Table 3). Their shared experiences with other members of the group made participants feel that the support they were receiving in this setting was more ‘genuine’ than that on offer from professionals or from family and/or friends. Many of the group members in both studies reported a reduction in self-harm following group membership. Participants described other positive changes that they attributed to group membership, including friendship and decreased isolation, and improvements in self-awareness, mood and interpersonal skills. They also reported that they derived a sense of empowerment and self-worth through witnessing and supporting each other's struggles and successes. Peer support group leaders reported positive experiences in relation to their sense of autonomy in running the group.^29^ These findings suggest that self-harm support groups are perceived by members as valuable peer support in helping to manage self-harm and are also acceptable to its members.

**Table 3:** Summary of the main benefits and risks identified in included studies

Main benefits	Strength of evidence^a^	Papers reporting this finding
Feeling accepted and understood Providing a safe space Feeling supported Giving support Providing a safe sense of community	10	Boyce et al (2018)^32^ Brow (2015)^25^ Corcoran et al (2017)^29^ Haberstroh & Moyer (2012)^31^ Lavis & Winter (2020)^26^ Murray & Fox (2006)^30^ Sharkey et al (2012)^28^ Smithson et al (2011)^27^ UTRIP (2012)^24^ Whitlock et al (2006)^33^
Decreased isolation, stigma and shame	4	Boyce et al (2018)^32^ Corcoran et al (2017)^29^ Haberstroh & Moyer (2012)^31^ Lavis & Winter (2020)^26^
Source of information on professional treatment	4	Brow (2015)^25^ Haberstroh & Moyer (2012)^31^ UTRIP (2012)^24^ Whitlock et al (2006)^33^
Reduced self-harm	4	Boyce et al (2018)^32^ Brow (2015)^25^ Corcoran et al (2017)^29^ Murray & Fox (2006)^30^
Source of information on self-harm	3	Lavis & Winter (2020)^26^ UTRIP (2012)^24^ Whitlock et al (2006)^33^
Sense of empowerment Increasing self-worth Increasing autonomy	2	Boyce et al (2018)^32^ Corcoran et al (2017)^29^
Reducing the risks of self-harm (harm minimisation)	1	Lavis & Winter (2020)^26^
Source of motivation for recovery	1	Brow (2015)^25^

Perceived risks	Strength of evidence^a^	Papers reporting this finding
Triggering self-harm (e.g. after viewing graphic images)	6	Brow (2015)^25^ Corcoran et al (2007)^29^ Haberstroh & Moyer (2012)^31^ Lavis & Winter (2020)^26^ Murray & Fox (2006)^30^ UTRIP (2012)^24^
Distress and burden from providing peer support to others	2	Lavis & Winter (2020)^26^ Smithson et al (2011)^27^ UTRIP (2012)^24^
Pressure to post extreme content or images to demonstrate a continued need for support from peers Disincentive to seeking ‘offline’ professional help	2	Lavis & Winter (2020)^26^ Whitlock et al (2006)^33^
Retraumatisation by triggers (e.g. stories)	1	Corcoran et al (2007)^29^
Learning new methods of self-harm	1	Whitlock et al (2006)^33^
Self-harm being normalised Reinforcement of self-harm to feel a sense of belonging	1	Whitlock et al (2006)^33^
Feelings or concerns ignored or invalidated	1	Smithson et al (2011)^27^

a. The number of included papers (out of ten) that reported this result.

#### Online peer support

Most studies of online moderated peer support investigated self-injury message boards or forums (*n* = 5/8).^25,27,28,30,33^ Of the remaining three studies, one explored an online recovery group consisting of individuals referred to the group by self-injury professionals;^31^ one investigated young people's engagement with self-harm content on social media such as Reddit, Instagram and Twitter;^26^ and one investigated online peer support services for young people who have self-harmed provided by a range of organisations across Denmark, Italy, Slovenia and the UK.^24^

Similar to the findings for face-to-face peer support, users of online peer support reported that their previous experiences of conventional (i.e. professional or non-peer) support were characterised by poor treatment and stigma, which drove them to seek alternative options.^26,31^ The key advantages perceived by participants in these online media included providing and receiving support from those with similar lived experiences.^26,30,31,33^ Advantages were also perceived in gaining access to useful information on self-harm,^24,26,33^ such as how to self-harm safely,^26^ how to conceal self-harm (i.e. methods of concealing cuts and scars) and how to seek treatment,^24,33^ and in having less anxiety around people finding out about the self-harming behaviour.^33^ In one study, the online group was also viewed as a useful supplement to counselling.^31^ However, a small proportion of participants (*n* = 12/74; 16%) in the study published by Murray & Fox (2006) reported that they used the forum with the intention of being triggered to self-harm.^30^ These participants described feeling competitive about their self-harm when reading posts, with some viewing their self-harm as ‘inadequate’ and some deliberately reading discussions when they felt the urge to self-harm.^30^

As no trials of online peer support met our inclusion criteria, effectiveness could not be established. In studies where participants were asked about the perceived effectiveness of online peer support in reducing their self-harm behaviours, 41–50% reported a decrease in self-harm, which they attributed to group membership.^25,30^ In the Murray & Fox (2006) study, 46% reported no change in their self-harm, and 10% reported an increase.^30^ The sense of safety and community stemming from online peer support made users feel supported by people who understood their experiences of self-harm, and less alone.^24,25,30,31^ Added perceived benefits included the sense that online peer support increased participants’ motivation to recover,^25^ prompted help-seeking for professional (i.e. formal) support,^25,31^ and could be accessed quickly and easily as a source of information.^24^

### Q2 and Q3. Risks and mitigation

#### Face-to-face peer support

Risks discussed in the Corcoran et al (2007) study included the potential for members to be re-traumatised through listening to each other's stories, as well as the risk of triggering self-injury through learning new methods within the group.^29^ The article included suggestions as to ways in which this could be mitigated, including the use of a professional facilitator who could establish clear and healthy boundaries within the group, as well as supplementing the group intervention with individual support.

#### Online peer support

Included articles identified three main risks perceived with online peer support interventions. First, the most frequently documented perceived risk identified from online peer support for self-harm was the risk of triggering self-harm behaviour.^24–26,31^ This could arise through: (a) unmoderated sharing of triggering content, such as graphic images, distressing stories, or new methods of self-harm or self-harm concealment methods;^31,33^ (b) having to prove continuously a need for help by posting more extreme content or images in order to sustain online peer support;^26^ (c) a desire to belong;^33^ or (d) the normalisation and reinforcement (sometimes seen as encouragement) of self-injurious behaviour.^33^ The second most common potential risk identified was that the use of these media could isolate members from the ‘offline’ world and hinder them from seeking professional and/or offline help.^26,33^ The third most common potential risk identified was that online peer support might have a negative impact on the well-being of peer supporters due to the distress and burden associated with hearing others’ stories and attempting to help,^24,26^ as well as feeling ignored^27^ or misunderstood, or being involved in a disagreement with other members.^24^ In addition, there was the potential for young people to feel overwhelmed while supporting others, as they may lack the skills or knowledge of how best to help.^24^

One way to mitigate these risks is to have an online moderator who enforces boundaries between members and overt ‘no triggering’ rules.^24,27,31^ However, moderators are sometimes individuals who self-harm or who have self-harmed, and there is a risk of this involvement being triggering for them too.^31^ Where moderators have lived experience of self-harm, it was suggested that they should be individuals who have established recovery and are well supported outside the online group.^24,31^ Some studies also highlighted the potential for clinicians to have an important adjunctive role in peer support for self-harm in two ways: clinicians may act as moderators where peer support is offered in clinical settings but should also inquire about patients’ engagement with peer support for self-harm offered online,^24,25^ so that they can support them in using forums constructively. It was also suggested that online media should signpost to offline resources to provide a safety net should other support be needed.^26^ One study also alluded to the potential benefits of smaller group size in online forums, which meant that users were less likely to feel ignored.^27^

## Discussion

The main findings of this systematic review were that only a few studies have investigated the provision, quality, acceptability or potential harms of peer interventions for self-harm, whether in the published or the grey literature. Of the studies available, none has evaluated the effectiveness of peer support for self-harm, which limits conclusions about its impact on key outcomes such as distress, stigma, depressive symptoms, suicidal ideation, suicide attempt and suicide, as well as objective measurement of the risk of potential adverse effects.

However, we did identify useful literature describing the perceptions of those who use peer support for self-harm in relation to its acceptability and potential harms. These studies described a range of perceived social benefits, including a reduction in loneliness and the gaining of a sense of community and interpersonal skills; they also described a range of emotional benefits including the sense of being able to help others, an opportunity to vent frustrations, and the provision of access to information and support. In addition, these studies described perceived clinical benefits, including a reduction in the frequency and severity of self-harm, improvements in mood, and an increase in the practice of safer methods of self-harm. No participants described feeling less suicidal as a consequence of using peer support for self-harm. However, this question would better addressed quantitatively using validated measures of suicidality. Many participants described how stigmatised and ashamed they had been made to feel when using more conventional sources of support for self-harm, contrasting this with their positive experiences of the community they had encountered through peer support.

Some important risks were also identified, including the potential for peer support to cause vicarious trauma or trigger self-harm behaviour in those listening to others’ stories or viewing others’ scars, as well as the potential for psychological processes such as reinforcement of self-harm, imitating others’ self-harming behaviours, comparing extent of injuries or self-harming in order to fit in with peers. There were also concerns expressed about the burden on those participating in peer support in having to support others despite not having appropriate training. Specific potential risks of online peer support were the difficulties of monitoring whether participants felt safe, and the potential for participants to rely on online support over formal sources of support, where this might otherwise be indicated. However, a number of valuable suggestions were made as to how these identified risks might be mitigated, including providing professional facilitators for groups and trigger warnings, and ensuring that peers who take on moderating roles feel well supported themselves.

### Strengths and limitations

Our systematic review had a number of key strengths that should reinforce the confidence of practitioners and policy makers when applying our findings in practice. We gained input from a researcher with lived experience, a mental health professional, a health psychologist and Samaritans when identifying search terms for this review and used these professional networks to contact key voluntary sector organisations and self-harm groups in order to identify grey literature. We also used Twitter for suggestions on published and unpublished literature on potential harms, in order to balance our study and counter potential publication bias. Our quality assessment of included studies used standardised tools, and the studies were judged to be of high quality. Our summary of the potential benefits and harms of peer support for self-harm presents a balanced account of the key considerations described in this published and unpublished literature when implementing peer support for self-harm, including key risks and mitigation recommendations.

In only searching two databases, we may have missed studies published in other journals, but our use of MEDLINE and PsycINFO was intended to focus our review on clinical findings. Our focus on non-clinical settings may have limited the lessons to be learned about other benefits of peer support for self-harm, but this specific focus was intended to address a research and policy gap in relation to non-clinical settings. We acknowledge that although our search of the published literature included international studies, our exclusion of non-English-language studies will have biased our report to reflect primarily the experiences of high-income countries. We also acknowledge that our search for grey literature reflected primarily UK-based organisations, given the location of the research team and funder, and this report might therefore be of less relevance to non-UK settings. We did not identify any trials, despite our comprehensive search terms, which meant that we could not present evidence of effectiveness. We also noted that the samples in included studies tended to underrepresent the experiences of people from minority ethnic groups; this may reflect sampling biases within those studies. We acknowledge that we may not have contacted the full range of experts in the field, who may have unpublished data not represented in this review, and we did not contact authors to clarify any queries over the presentation of data.

In this review, our exclusive focus on peer support for self-harm may have neglected a wider perspective in which peer support is compared directly with other forms of support for self-harm in terms of relative acceptability. The findings of an Australian survey of young people who had self-harmed is particularly striking in this respect.^34^ This study aimed to explore the attitudes of young people who had self-harmed towards the use of online help for self-injury, as a means of informing future service delivery. Survey responses from 457 young people who had self-injured identified preferences for future online help-seeking, the rationale for which included gaining information and guidance, reducing isolation, a preference for an online culture, facilitation of help-seeking, easy access to support and the advantages of privacy. Of all sources of online support listed (e.g. texting, gaming, direct links to professionals, self-help and peer support) the most popular option was contact with a professional via instant messaging. Professional help therefore appeared to be preferred to peer support within the online context, highlighting the importance of considering hybrid sources of support.

### Future research

The main gaps in research that we identified were studies describing the effectiveness or cost-effectiveness of peer support for self-harm, as we did not identify any trials or economic analyses. The published and unpublished studies we found suggested that peer support for self-harm is an acceptable approach for a specific subset of people who have self-harmed, and that they and the professionals who support them show a strong awareness of the potential risks and mitigations required in order to provide a safe service. Although this review identified the triggering of self-harm as being the most common potential risk attached to peer support, no studies evaluated whether the benefits of peer support interventions for self-harm outweigh the risks. Clinical trials and large-scale observational studies are required to measure both positive and adverse effects so we can build on our subjective understanding of peer support for self-harm. We also need cost-effectiveness analyses that take a wide societal perspective, taking into account the potential for clinical benefits and adverse effects, and the costs and benefits to carers, the health service, and emergency services, as well as use of other support and treatment options.

We identified no study describing the geographical provision of peer support for self-harm, nor any overview of current online provision. These are is needed to ascertain whether there are disparities in provision for certain geographical areas or for certain digitally excluded groups. In particular, we hope that future studies will explore the acceptability of peer support for people from ethnic minorities who self-harm, and that the lack of trial evidence might be addressed through controlled trials and the publication of their results. We recommend that regular updates of this review will inform updated recommendations on the provision, quality, acceptability, effectiveness and potential harms of peer support for self-harm.

### Policy implications

These findings suggest that the provision of peer support for self-harm is acceptable and valued by some people who have self-harmed, but that participants also perceived specific risks and ways to mitigate these. The range of benefits described suggests that there would be value in implementing further peer support services for people who have self-harmed, provided that service planning includes a careful consideration of risk management. The generally young age of the samples described in this review, typically 16 to 25 years, suggests that this would be welcomed by adolescents who self-harm, given their general preference for peer support over formal support.^8^ The stigma and shame experienced when accessing professional support was contrasted with the more accepting experience of using peer support. This has important policy implications, given that stigma has been identified as a key barrier to seeking professional support for mental health problems among young people.^35^ Online peer support was viewed positively as a way of promoting help-seeking for professional support and may be a critical stage in the process of recognising a need for support and identifying the most acceptable routes into professional support.

The suggestions made around risk mitigation processes suggest a key role for training and supporting group facilitators and online group moderators, both in relation to the processes they follow when providing peer support (policies on triggering content and ground rules about expectations in showing respect in interactions) but also in relation to supporting their emotional needs (Box 1). The provision of regular supervision may well be welcomed by those providing support, as well as risk management procedures should they feel overwhelmed by responsibilities and clinical risk scenarios. All those who take part in a peer support group may at times feel overwhelmed, and it may be important to consider ground rules on taking a break from the group or accessing alternative sources of support. Improving the confidence of people who have self-harmed to support vulnerable others might be achieved through interventions such as Mental Health First Aid Training to improve mental health literacy.^36^ Peer support services might also consider providing a set of guidelines on how peers can best support others, including how to maintain one's own emotional health in order to be in the best place to help others. More generally, it will also be important when implementing peer support to provide clear signposting to other sources of support, should these be indicated alongside engagement with a peer community.

Box 1 Recommendations for commissioning online peer support groups for self-harm.

(a) Ensure there are moderators for online peer support forums

(b) Ensure that moderators are well supported outside the online group

(c) Suggest use of mental health professionals as moderators for online peer support forums

(d) Supplement peer group support with other sources of support (including formal sources of support)

(e) Ensure that members are signposted to offline sources of support

The important part that peer support plays in the lives of some people who have self-harmed suggests that this is an important dimension of a clinical assessment, and that clinicians should inquire routinely about peer support when taking a clinical history from a person who self-harms, particularly that gained online.^37^ Given the acceptability of peer support to people who have self-harmed, clinicians should also become familiar with the services available and discuss these as part of care planning alongside a consideration of the potential risks described. This is particularly important during a period of pandemic restrictions, when access to a full range of support sources may be severely limited. There may also be a role for clinicians in supporting the moderators or facilitators of peer support, given the importance of risk protocols and risk management when offering this type of help to a vulnerable group.

## Conclusions

Our review of the literature suggests that peer support for self-harm has an important therapeutic role in the lives of some people who have self-harmed, who describe social, emotional and clinical benefits but are also able to recognise the potential for harms. This literature provides valuable suggestions for how to best implement peer support for self-harm, whether face-to-face or online, and the risk issues that that need to be considered in order to provide this safely. In view of the preferences of young people for self-harm support outside formal healthcare settings, peer support for self-harm could be a very valuable means of containing some of the distress and loneliness associated with self-harm and promoting a sense of autonomy and community.

### Lived experience commentary

The significance of providing peer support for those who self-harm is evidential; the current review summarises that although it is not without its risks, peer support both face-to-face and via online media can help some young people manage self-injurious thoughts and behaviours. Through open discussions around self-harm via in-person or virtual support groups, young people have reported a decrease in perceived stigma and shame compared with interventions led by professionals or loved ones. This may be attributed to a sense of solidarity, whereby those who self-harm are provided with a safe space to share their narratives and support each other's struggles and goals, during times where they may feel isolated and misunderstood by those who do not share similar experiences.

From my perspective, this review has brought to light how society has altered the way young people choose to obtain informal support. Although face-to-face groups have their advantages, it is interesting to note that online platforms are an increasingly popular means for seeking help, owing to the opportunities for giving/receiving support from a range of like-minded people, and the instant access to useful information. These findings strongly resonate with my own lived experience as an adolescent; I found solace in the mutual benefits of sharing emotional distress and self-harm ideation with others, while maintaining anonymity behind a mobile screen. However, the associated risks of using online media should not go unnoticed; these include exposure to graphic images of extreme self-harm and feeling overwhelmed with a perceived responsibility to support others.

Although I understand the high-risk nature of peer support in increasing some self-harm behaviours, I consider the benefits to outweigh the cons in terms of accessing support and generating a sense of self-empowerment and online community presence for young people. Given these potential benefits, and in light of the few studies investigating the effectiveness of peer interventions, there is an urgent need to determine the efficacy of peer support as an active intervention. Only through clinical trials can risk issues concerning both group members and facilitators/moderators be fully addressed, and peer interventions could start to be considered as having the same necessity for managing self-harm as professional support services.

## Data Availability

Data availability is not applicable to this article as no new data were created or analysed in this study.

## Supplementary material

For supplementary material accompanying this paper visit http://doi.org/10.1192/bjo.2021.1081. click here to view supplementary material
