# Supplementary material for: Effectiveness, acceptability and potential harms of peer support for self-harm in non-clinical settings: systematic review
Source: BJPsych Open. 2022 Jan 17;8(1):e28. doi: 10.1192/bjo.2021.1081 (PMC8811789; doi:10.1192/bjo.2021.1081)
Supplement: Supplementary file 1 [file S2056472421010814sup001.docx]

**Supplementary material**

**1. Search Strategies**

1A. MEDLINE

1. self-injurious behavior/ or self mutilation/ (11249)
2. (parasuicide or "suicidal self-injur" or "suicidal self?injur*" or SSI or "self-injurious behavi*" or "self?injurious behavi*" or "suicidal behavi*" or automutilation or auto-mutilation or "auto?mutilation" or self-mutilation or "self?mutilation" or "self-inflicted wound*" or "self?inflicted wound*" or "self-inflicted injur*" or "self?inflicted injur*" or "non-suicidal self-injur*" or "non-suicidal self? injur*" or NSSI).tw. (21766)
3. (self-harm* or "self?harm*" or self-injur* or "self?injur*" or self-destruct* or "self?destruct*" or self-poison* or "self?poison*" or self-immolat* or "self?immolat*" or self-inflict* or "self?inflict*" or auto-destruct* or "auto?destruct*" or (self adj2 cut*) or cut*).tw. (439664)
4. 1 or 2 or 3 (459740)
5. peer group/ or Social support/ or Self-help groups/ or Education, Nonprofessional/ or Psychosocial support systems/ or Community networks/ (105183)
6. (((peer* or support* or volunteer* or "lived experience" or "service user*" or community or lay or mutual) adj2 (group* or network* or communit* or relation* or support* or listen* or help* or visit* or aid*)) or ((social or community) adj3 (support or network* or group*)) or ((peer* or lay or volunteer *) adj (based or counsel* or deliver* or interact* or led or mediat* or operat* or provid* or run*)) or "peer support" or peer-to-peer or "peer?to?peer" or peer* or "social interaction program*" or befriend*).tw. (2147753)
7. (((lay or peer) adj2 (advisor* or consultant* or educator* or expert* or facilitator* or instructor* or leader* or person* or tutor* or worker* or advice* or advise* or counsel* or mentor*)) or "expert patient*").tw. (6624)
8. 5 or 6 or 7 (2181210)
9. ("facebook" or e-mail or e-support or "e-bulletin board" or e-society or e-community or forum or "discussion group" or "message board" or "internet relay" or "instant?messaging" or "chat technology" or "chat room" or chatroom or chatgroup or "chat group" or ((virtual or internet or online or web-based or "web?based" or internet-based or "internet?based") adj2 (support or network or group or society or community or interaction or "bulletin board"))).tw. (30668)
10. (helpline or "help?line" or ((phone* or telephone*) adj3 (help* or instruct* or interact* or mediat* or program* or rehab* or strateg* or support* or teach* or train* or workshop* or based or assist* or driven or led))).tw. (11366)
11. 9 or 10 (41824)
12. 1 ("peer?support" or peer-support or "self?help" or self-help or "social support" or "support group" or "support network" or "support system").tw. (62443)
13. 11 and 12 (2371)
14. 8 or 13 (2181336)
15. 4 and 14 (36840)
16. limit 15 to (english language and humans and yr="2000 -Current") (19899)

1B. PsycINFO

1. self-injurious behavior/ or self mutilation/
2. (parasuicide or "suicidal self-injur" or "suicidal self?injur*" or SSI or "self-injurious behavi*" or "self?injurious behavi*" or "suicidal behavi*" or automutilation or auto-mutilation or "auto?mutilation" or self-mutilation or "self?mutilation" or "self-inflicted wound*" or "self?inflicted wound*" or "self-inflicted injur*" or "self?inflicted injur*" or "non-suicidal self-injur*" or "non-suicidal self? injur*" or NSSI).tw. (17882)
3. (self-harm* or "self?harm*" or self-injur* or "self?injur*" or self-destruct* or "self?destruct*" or self-poison* or "self?poison*" or self-immolat* or "self?immolat*" or self-inflict* or "self?inflict*" or auto-destruct* or "auto?destruct*" or (self adj2 cut*) or cut*).tw. (58008)
4. 1 or 2 or 3 (69788)
5. peer group/ or Social support/ or Self-help groups/ or Education, Nonprofessional/ or Psychosocial support systems/ or Community networks/ (37486)
6. (((peer* or support* or volunteer* or "lived experience" or "service user*" or community or lay or mutual) adj2 (group* or network* or communit* or relation* or support* or listen* or help* or visit* or aid*)) or ((social or community) adj3 (support or network* or group*)) or ((peer* or lay or volunteer *) adj (based or counsel* or deliver* or interact* or led or mediat* or operat* or provid* or run*)) or "peer support" or peer-to-peer or "peer?to?peer" or peer* or "social interaction program*" or befriend*).tw. (998301)
7. (((lay or peer) adj2 (advisor* or consultant* or educator* or expert* or facilitator* or instructor* or leader* or person* or tutor* or worker* or advice* or advise* or counsel* or mentor*)) or "expert patient*").tw. (7396)
8. 5 or 6 or 7 (1000384)
9. ("facebook" or e-mail or e-support or "e-bulletin board" or e-society or e-community or forum or "discussion group" or "message board" or "internet relay" or "instant?messaging" or "chat technology" or "chat room" or chatroom or chatgroup or "chat group" or ((virtual or internet or online or web-based or "web?based" or internet-based or "internet?based") adj2 (support or network or group or society or community or interaction or "bulletin board"))).tw. (24369)
10. (helpline or "help?line" or ((phone* or telephone*) adj3 (help* or instruct* or interact* or mediat* or program* or rehab* or strateg* or support* or teach* or train* or workshop* or based or assist* or driven or led))).tw. (6334)
11. 9 or 10 (30591)
12. ("peer?support" or peer-support or "self?help" or self-help or "social support" or "support group" or "support network" or "support system").tw. (71602)
13. 11 and 12 (2310)
14. 8 or 13 (1000529)
15. 4 and 14 (15315)
16. limit 15 to (human and english language and yr="2000 -Current") (11768)

1C. Open Grey

- Self injury  and social support
- self injury and self help groups
- self injury and psychosocial support
- self injury and community networks
- self harm AND peer group
- self harm AND social support
- Self harm and self help group
- Self harm and lived experience
- self harm and volunteer
- Self harm and lay expert
- suicidal self injury and peer support
- suicidal self injury and social support
- non- suicidal self injury and social support
- non- suicidal self injury and peer support
- NSSI and social support
- NSSI and peer support
- parasuicide and support group
- parasuicide and peer group
- parasuicide and peer support
- self injury and volunteer
- self injury and lay expert
- Self mutilation and peer group
- Self injurious and ‘peer group’

1. Websites searched and organisations contacted for grey literature search

| - We Are with You (formerly known as Addaction) - Mind - Mental Health Foundation - Rethink Mental Illness - Time to Change - Together for Mental Wellbeing - Harmless - Young Minds - Agenda - Beat - Anorexia, Bulimia and Care (ABC) - Overeaters Anonymous - Alcoholics Anonymous - Narcotics Anonymous - Frank |
| --- |

3. Quality Appraisal

Table 3A: Quality appraisal of qualitative studies using the Critical Appraisals Skill Programme (CASP) Qualitative Checklist

| **Study** | **Was there a clear statement of the aims of the research?** | **Is a qualitative methodology appropriate?** | **Was the research design appropriate to address the aims of the research?** | **Was the recruitment strategy appropriate to the aims of the research?** | **Was the data collected in a way that addressed the research issue?** | **Has the relationship between researcher and participants been adequately considered?** | **Have ethical issues been taken into consideration?** | **Was the data analysis sufficiently rigorous?** | **Is there a clear statement of findings?** | **How valuable is the research?** | **Score out of 10** |
| --- | --- | --- | --- | --- | --- | --- | --- | --- | --- | --- | --- |
| Boyce, Munn-Giddings & Seckler (2018) | Yes | Yes | Yes | Yes | Yes | No | No | Yes | Yes | Valuable | 8 |
| Brow (2015) | Yes | Yes | Yes | Yes | Yes | Can’t tell | Yes | Yes | Yes | Valuable | 9 |
| Corcoran, Mewse & Babiker  (2007) | Yes | Yes | Yes | Yes | Yes | Can’t tell | Yes | Yes | Yes | Valuable | 9 |
| Haberstroh & Moyer (2012) | Yes | Yes | Yes | Yes | Yes | Can’t tell | Yes | Yes | Yes | Valuable | 9 |
| Lavis & Winter   (2020) | Yes | Yes | Yes | Yes | Yes | Can’t tell | Yes | Can’t tell | Yes | Valuable | 8 |
| Murray & Fox (2006) | Yes | Yes | Yes | Yes | Can’t tell | Can’t tell | Yes | Yes | Yes | Valuable | 8 |
| Sharkey et al. (2012) | Yes | Yes | Yes | Yes | Yes | Can’t tell | Yes | Yes | Yes | Valuable | 9 |
| Smithson et al. (2011) | Yes | Yes | Yes | Yes | Yes | Can’t tell | Yes | Yes | Yes | Valuable | 9 |

Table 3B: Quality appraisal of mixed methods studies using the Mixed Methods Appraisal Tool (MMAT)

| **Study** | **Qualitative** | | | | | **Quantitative** | | | | | **Mixed Methods** | | | | | **Score out of 15** |
| --- | --- | --- | --- | --- | --- | --- | --- | --- | --- | --- | --- | --- | --- | --- | --- | --- |
|  | **1.1** | **1.2** | **1.3** | **1.4** | **1.5** | **4.1** | **4.2** | **4.3** | **4.4** | **4.5** | **5.1** | **5.2** | **5.3** | **5.4** | **5.5** |  |
| Whitlock et al. (2006) | Yes | Yes | Yes | Yes | Yes | Yes | Can’t tell | Can’t tell | Can’t tell | Yes | Yes | Yes | Yes | Yes | Can’t tell | 11 |

Table 3C: Quality appraisal of reports not presented as primary research studies using NESTA’s Standards of Evidence

| **Study** | **Level 1: Have they described what positive difference this innovation is going to make?** | **Level 2: Is there data that shows the positive difference can be measured?** | **Level 3: Have they proved that the positive difference is because of the innovation (by testing with comparison groups)?** | **Level 4: Have other people proved the same thing?** | **Level 5: Can this innovation be replicated elsewhere?** | **Levels achieved** |
| --- | --- | --- | --- | --- | --- | --- |
| Institute for Research and Development “UTRIP” (2012) | Yes | Yes | Not sure | Yes | Yes | 2 |

| **Section and Topic** | **Item #** | **Checklist item** | **Location where item is reported** |
| --- | --- | --- | --- |
| **TITLE** | | |  |
| Title | 1 | Identify the report as a systematic review. | 1 |
| **ABSTRACT** | | |  |
| Abstract | 2 | See the PRISMA 2020 for Abstracts checklist. | 2-3 |
| **INTRODUCTION** | | |  |
| Rationale | 3 | Describe the rationale for the review in the context of existing knowledge. | 4 |
| Objectives | 4 | Provide an explicit statement of the objective(s) or question(s) the review addresses. | 5 |
| **METHODS** | | |  |
| Eligibility criteria | 5 | Specify the inclusion and exclusion criteria for the review and how studies were grouped for the syntheses. | 7-9 |
| Information sources | 6 | Specify all databases, registers, websites, organisations, reference lists and other sources searched or consulted to identify studies. Specify the date when each source was last searched or consulted. | 6 |
| Search strategy | 7 | Present the full search strategies for all databases, registers and websites, including any filters and limits used. | 47-50 |
| Selection process | 8 | Specify the methods used to decide whether a study met the inclusion criteria of the review, including how many reviewers screened each record and each report retrieved, whether they worked independently, and if applicable, details of automation tools used in the process. | 8 |
| Data collection process | 9 | Specify the methods used to collect data from reports, including how many reviewers collected data from each report, whether they worked independently, any processes for obtaining or confirming data from study investigators, and if applicable, details of automation tools used in the process. | 8-9 |
| Data items | 10a | List and define all outcomes for which data were sought. Specify whether all results that were compatible with each outcome domain in each study were sought (e.g. for all measures, time points, analyses), and if not, the methods used to decide which results to collect. | 9 |
|  | 10b | List and define all other variables for which data were sought (e.g. participant and intervention characteristics, funding sources). Describe any assumptions made about any missing or unclear information. | 8-9 |
| Study risk of bias assessment | 11 | Specify the methods used to assess risk of bias in the included studies, including details of the tool(s) used, how many reviewers assessed each study and whether they worked independently, and if applicable, details of automation tools used in the process. | 9-10 |
| Effect measures | 12 | Specify for each outcome the effect measure(s) (e.g. risk ratio, mean difference) used in the synthesis or presentation of results. |  |
| Synthesis methods | 13a | Describe the processes used to decide which studies were eligible for each synthesis (e.g. tabulating the study intervention characteristics and comparing against the planned groups for each synthesis (item #5)). | 9-10 |
|  | 13b | Describe any methods required to prepare the data for presentation or synthesis, such as handling of missing summary statistics, or data conversions. | 9-10 |
|  | 13c | Describe any methods used to tabulate or visually display results of individual studies and syntheses. |  |
|  | 13d | Describe any methods used to synthesize results and provide a rationale for the choice(s). If meta-analysis was performed, describe the model(s), method(s) to identify the presence and extent of statistical heterogeneity, and software package(s) used. | n/a |
|  | 13e | Describe any methods used to explore possible causes of heterogeneity among study results (e.g. subgroup analysis, meta-regression). | n/a |
|  | 13f | Describe any sensitivity analyses conducted to assess robustness of the synthesized results. | n/a |
| Reporting bias assessment | 14 | Describe any methods used to assess risk of bias due to missing results in a synthesis (arising from reporting biases). |  |
| Certainty assessment | 15 | Describe any methods used to assess certainty (or confidence) in the body of evidence for an outcome. |  |
| **RESULTS** | | |  |
| Study selection | 16a | Describe the results of the search and selection process, from the number of records identified in the search to the number of studies included in the review, ideally using a flow diagram. | 12 |
|  | 16b | Cite studies that might appear to meet the inclusion criteria, but which were excluded, and explain why they were excluded. |  |
| Study characteristics | 17 | Cite each included study and present its characteristics. | 34-45 |
| Risk of bias in studies | 18 | Present assessments of risk of bias for each included study. | 51-52 |
| Results of individual studies | 19 | For all outcomes, present, for each study: (a) summary statistics for each group (where appropriate) and (b) an effect estimate and its precision (e.g. confidence/credible interval), ideally using structured tables or plots. | n/a |
| Results of syntheses | 20a | For each synthesis, briefly summarise the characteristics and risk of bias among contributing studies. | 14-18 |
|  | 20b | Present results of all statistical syntheses conducted. If meta-analysis was done, present for each the summary estimate and its precision (e.g. confidence/credible interval) and measures of statistical heterogeneity. If comparing groups, describe the direction of the effect. | n/a |
|  | 20c | Present results of all investigations of possible causes of heterogeneity among study results. | n/a |
|  | 20d | Present results of all sensitivity analyses conducted to assess the robustness of the synthesized results. | n/a |
| Reporting biases | 21 | Present assessments of risk of bias due to missing results (arising from reporting biases) for each synthesis assessed. | n/a |
| Certainty of evidence | 22 | Present assessments of certainty (or confidence) in the body of evidence for each outcome assessed. | 19-20 |
| **DISCUSSION** | | |  |
| Discussion | 23a | Provide a general interpretation of the results in the context of other evidence. |  |
|  | 23b | Discuss any limitations of the evidence included in the review. | 22-24 |
|  | 23c | Discuss any limitations of the review processes used. | 22-24 |
|  | 23d | Discuss implications of the results for practice, policy, and future research. | 24-26 |
| **OTHER INFORMATION** | | |  |
| Registration and protocol | 24a | Provide registration information for the review, including register name and registration number, or state that the review was not registered. |  |
|  | 24b | Indicate where the review protocol can be accessed, or state that a protocol was not prepared. | 5 |
|  | 24c | Describe and explain any amendments to information provided at registration or in the protocol. |  |
| Support | 25 | Describe sources of financial or non-financial support for the review, and the role of the funders or sponsors in the review. | 30 |
| Competing interests | 26 | Declare any competing interests of review authors. | 30 |
| Availability of data, code and other materials | 27 | Report which of the following are publicly available and where they can be found: template data collection forms; data extracted from included studies; data used for all analyses; analytic code; any other materials used in the review. |  |
